# Eosinophil extracellular traps in eosinophilic chronic rhinosinusitis induce Charcot–Leyden crystal formation and eosinophil recruitment

**DOI:** 10.1042/BSR20230410

**Published:** 2024-03-01

**Authors:** Siyuan Zhang, Zhenlin Wang

**Affiliations:** Department of Otorhinolaryngology Head and Neck Surgery, Xuanwu Hospital, Capital Medical University, Beijing, China

**Keywords:** Charcot-Leyden crystals, Eosinophilic chronic rhinosinusitis, Eotaxin-3, Extracellular eosinophilic traps

## Abstract

Eosinophil extracellular traps (EETs) are implicated in various eosinophil-associated diseases; however, their role in chronic rhinosinusitis (CRS) remains unclear. In the present study, 57 CRS patients were enrolled, and immunofluorescence was used to analyze EETs in eosinophilic (eCRS) and non-eosinophilic (Non-eCRS) tissues. MSD was used to examine IL-4, IL-5, and IL-13 concentrations in tissue homogenates. Charcot–Leyden crystals (CLCs) protein expression was detected in PMA, PMA+DNase I, and blank control eosinophils using ELISA. Eotaxin-3 mRNA and protein levels were measured in human nasal epithelial cells (HNECs) cultured with EETs, EETs+DNase I, DNase I, and unstimulated eosinophils using PCR and ELISA. EETs were significantly increased in eCRS tissues compared with Non-eCRS (*P*<0.001), and correlated with VAS and Lund–Mackay CT scores. IL-5 expression was related to EETs formation (*r* = 0.738, *P*<0.001). PMA-stimulated eosinophils exhibited higher CLCs protein levels (*P*<0.01). Co-culturing HNECs with EETs significantly increased eotaxin-3 mRNA and protein levels (*P*<0.0001, *P*<0.001) compared with other groups. The study suggests EETs formation is elevated in eCRS patients and is involved in CLCs formation and chemokine secretion, promoting eosinophilic inflammation.

## Introduction

Chronic rhinosinusitis (CRS) is a persistent inflammatory condition of the nasal and sinus cavities characterized by nasal congestion, rhinorrhea, facial pain, and reduced sense of smell. It is a common disease, affecting 5–12% of the population in Western countries. CRS can be divided into two subtypes based on the presence or absence of nasal polyps: chronic rhinosinusitis with nasal polyps (CRSwNP) and chronic rhinosinusitis without nasal polyps (CRSsNP) [1]. CRSwNP is frequently linked to type 2 inflammation, an immune response mediated by Th2 cells and characterized by the production of cytokines like interleukin (IL)-4, IL-5, and IL-13. Eosinophilic chronic rhinosinusitis (eCRS) and non-eosinophilic chronic rhinosinusitis (Non-eCRS) are two recently defined CRS subtypes, based on the extent of eosinophilic infiltration in the sinus mucosa [2]. eCRS patients often clinically present with worse symptoms and treatment effects are not satisfactory compared with non-eCRS. Nevertheless, some patients may continue to experience recurring symptoms despite receiving maximum medical therapy and undergoing endoscopic sinus surgery.

Eosinophils produce granule proteins that can be harmful to both external pathogens and host tissue. Eosinophils release these granules through classical exocytosis, compound exocytosis, and piecemeal degranulation. The new phenomenon of eosinophils releasing extracellular traps was first introduced in 2008, four years after the discovery of neutrophils extracellular trap (NETs) [3]. This process of eosinophil cell death in which eosinophil extracellular traps (EETs) are released is known as EETosis. EETs are comprised of DNA and granule proteins, and their formation is often accompanied by the development of Charcot–Leyden crystals (CLCs) [4]. Therefore, EETosis may perpetuate eosinophilic inflammation even after the demise of short-lived eosinophils.

While the production of EETs is known to contribute to host defense, they have also been implicated in various eosinophil-associated diseases, including CRSwNP, eosinophilic esophagitis, and allergic asthma [5]. Ueki et al. found that EETs are present in CRSwNP secretions and contribute to increased viscosity [6]. EETs were concentrated in areas where *Staphylococcus aureus* was detected and in the subepithelial region, trapping the pathogen exclusively in regions of epithelial damage [7]. Galectin-10 has been shown to undergo a crystallization process mediated by EETosis, which involves changes in intracellular localization and increased extracellular concentration of CLCs [4]. Furthermore, Bachert et al. demonstrated that CLCs can stimulate epithelial cells to recruit neutrophils and produce cytokines such as GM-CSF, TNF-α, and IL-1, which can activate neutrophils and potentially enhance their effector function [8]. However, there is limited direct evidence *in vitro* regarding the significance of EETs in CRS. NETs can stimulate human nasal epithelial cells (HNECs) to release cytokines, including IL-8 and eotaxin, which are involved in the recruitment and migration of inflammatory cells such as neutrophils and eosinophils [9]. Notably, previous reports consistently indicated an up-regulation of eotaxin-3 expression in nasal polyp (NP) tissues relative to healthy controls, with this expression predominantly derived from epithelial cells. Eotaxin-3 has been shown to induce the chemotaxis of eosinophils, basophils, and Th2 lymphocytes, thereby contributing to eosinophilic inflammation [10]. Based on these findings, we propose that increased levels of EETs in NP tissues may upregulate the expression of eotaxin-3 and promote its secretion, ultimately exacerbating eosinophilic inflammation. DNase I, which disintegrates the chromatin in EETs, has shown potential as a method for inhibiting EETs formation and activity [3]. Moreover, we also investigated whether inhibiting EETs formation could be an efficacious strategy for CRS treatment.

## Materials and methods

### Subjects and data collection

The study enrolled 57 patients with chronic rhinosinusitis (CRS) who met the criteria outlined in the European Position Paper on Rhinosinusitis and Nasal Polyps (EPOS2020). The diagnosis of eCRS was made if the tissue eosinophil count was equal to or greater than ten per high-power field (400×), while non-eCRS was diagnosed if the count was less than ten per high-power field (400×) [2]. Demographic data were collected through face-to-face questionnaire interviews. Clinical data were obtained from patients’ medical records before operation. The age, sex, polyps, asthma and peripheral blood eosinophils were recorded from each participant. The presence of nasal polyps was determined based on nasal endoscopy. Asthma diagnosis was confirmed by a pulmonary physician according to Global Initiative for Asthma (GINA) guideline. We assessed the clinical symptoms with visual analog scale (VAS), endoscopy scores (Lund–Kennedy), CT scores (Lund–Mackay) [2]. In the study, the VAS was employed to assess participants’ perceptions of their current health status regarding CRS. Participants were instructed to indicate their perceived health status by placing a mark on a 10 cm line. The scale was anchored at 0, signifying the ‘perfect health’, and at 10, representing ‘worst imaginable health’. The location of the participant’s mark was then measured to quantify their health perception. The study was approved by the ethical committees of Xuanwu Hospital, Capital Medical University, and all study participants provided informed consent. Tissue samples were collected from patients undergoing endoscopic nasal surgery in the Department of Otolaryngology-Head and Neck Surgery, Xuanwu Hospital, Capital Medical University between October 2020 and October 2021.

### Hematoxylin and eosin staining

The nasal tissue samples were first fixed in 4% paraformaldehyde and then embedded in paraffin for further analysis. The tissue was sliced into 4 μm thick coronal sections, which were then stained with hematoxylin and eosin. Bright-field light microscopy (Olympus, Tokyo, Japan) was used to examine five non-overlapping fields of the subepithelial layer, and the number of eosinophils and total inflammatory cells per high-power field (HPF; ×400) was determined. Two independent pathologists, blinded to the patients’ clinical diagnosis and characteristics, evaluated all stained samples. The mean number of eosinophils was calculated based on their observations.

### Immunofluorescence

EETs were identified as previously described [11,12]. Briefly, nasal tissue samples were fixed in 4% paraformaldehyde and embedded in paraffin. EETs were visualized in 4-μm-thick sections by indirect immunofluorescence, followed by counterstaining for DNA. The sections were blocked using 1% bovine serum albumin (BSA) (Solarbio, Beijing, China), and then incubated with anti-major basic protein (MBP) polyclonal antibody (Santa Cruz, California, U.S.A.). TRITC from (ZSGB-BIO, Beijing, China) was used for secondary incubation, while DNA was visualized with DAPI (Solarbio, Beijing, China). The slides were scanned using the Panoramic 250 Flash Slide Scanner (3DHISTECH, Budapest, Hungary). EETs were counted in five non-overlapping fields of the subepithelial layer per high power field (HPF; ×400) for each patient, and the mean number of EETs was calculated. EET-positive cells were identified by the presence of extracellular, decondensed DNA in fibers or a web-like shape colocalized with MBP. All stained samples were observed by two independent pathologists blinded to the clinical diagnosis and patient characteristics.

### Real-time PCR

Total RNA was extracted from either frozen tissue or cultured cells using TriQuick reagent (Solarbio, Beijing, China), followed by reverse transcription using M-MLV Reverse Transcriptase (Promega, Wisconsin, U.S.A.). Two micrograms of total RNA were reversely transcribed into cDNA, which served as the template for real-time PCR. The mRNA expression of CLCs and eotaxin-3 was detected using SYBR Green PCR Mastermix (Solarbio, Beijing, China) on a LightCycler480 (Roche, Penzberg, Germany). The primers (Tsingke Biotech, Beijing, China) were as follows: for CLCs (133 bp), forward primer 5′-TCTACTGTGACAATCAAAGGGC-3′ and reverse primer 5′-CACGACGACCAAAGCACAC-3′; for eotaxin-3 (128 bp), forward primer 5′-ACGGGTACATGCCTAGGAGT-3′ and reverse primer 5′-AGTGAGGATGTGGTGCATGG-3′. The internal control GAPDH (112 bp) primers were forward primer, 5-CGGAGTCAACGGATTTGGTC-3, and reverse primer, 5-GGGTGGAATCATATTGGAACAT-3. The comparative Ct method (2^−ΔΔCt^) was used for relative gene expression analysis.

### Eosinophil purification and activation

Peripheral blood eosinophils were purified from eight healthy individuals, 4 ml of each people was collected in EDTA-containing tubes. The blood samples were diluted and slowly layered on to 8 ml of Lymphocyte Separation Medium (Tbd science, Tianjin, China) before centrifugation at 500 ***g*** for 20 min at room temperature without interruption. The resulting cell pellet was resuspended in 10 ml of NH_4_Cl for 10 min, followed by centrifugation at 500 ***g*** for 5 min at 4°C. The supernatant was discarded, and the cell pellet was retained. The granulocyte-enriched fractions were incubated with a biotinylated antibody cocktail and anti-biotin microbeads, then passed through magnetized columns (Miltenyi Biotec, California, U.S.A.). Non-eosinophils were retained in MACS® columns placed in a MACS® separator (Miltenyi Biotec, California, U.S.A.), while eosinophils passed through the columns and were collected as the enriched, unlabeled cell fraction. The purified eosinophils were cultured in RPMI 1640 (Solarbio, Beijing, China) in 0.1% FBS (Gibco, California, U.S.A.). Eosinophil purity was assessed by flow cytometric analysis and found to be over 90%.

EETs were induced by PMA and isolated as described previously [4,13]. To induce EETs formation, eosinophils were primed for 3 h with 10 ng/ml PMA (Solarbio, Beijing, China) to promote EET formation. Following stimulation, the medium was discarded, and the adherent layer was rinsed with PBS. The supernatant was collected 30 min after treatment with 1 U/ml MNase (Thermo Fisher Scientific, Massachusetts, U.S.A.) by centrifugation at 2500 ***g*** for 5 min. After removing cells and cell debris by centrifugation, supernatants containing EETs were harvested. Isolated EETs were stored at −80°C within seven days for future research.

Isolate eosinophils from peripheral blood (2 × 10^5^/200 μl) and seed them in a 24-well plate. They are divided into three groups, with three wells per group, and the following components are added to each group: 10 ng/ml PMA, 10 ng/ml PMA+10 U/ml DNase I, no additional components are added. Incubate the seeded cells in a 37°C incubator with 5% CO_2_ for 180 min.

### HNECs culture and stimulation

Five CRSwNP patients’ polyp tissues obtained during surgery were immediately placed in cold Hank’s balanced salt solution (HBSS). The samples were washed six times in cold phosphate-buffered saline (PBS) supplemented with 100 U/ml penicillin, 100 mg/ml streptomycin, and 25 µg/ml amphotericin B. The tissue samples were then enzymatically dissociated by incubating them in 0.1% Proteinase K (Solarbio, Beijing, China) in DMEM (Sigma-Aldrich, Missouri, U.S.A.) overnight at 4°C. At the end of the incubation, 5.0% FBS (Gibco, California, U.S.A.) was added to the medium to stop the enzymatic digestion and release the epithelial cells from the tissue by vigorous shaking. The cell suspension was centrifuged at 1000 rpm for 5 min, and the dissociated cells were plated on plastic culture dishes for 1 h at 37°C to eliminate fibroblasts. The epithelial cell suspension was then seeded on to 6.5-mm-diameter Polycarbonate membrane transwell with a pore size of 0.4 μl (Corning, New York, U.S.A.), at a density of 100,000 cells per well, to establish submerged cell cultures in BEGM (Lonza, Basel, Switzerland). The cells were incubated at 37°C in 5% CO_2_, and the culture medium was changed every 48 h. Once the cells had grown to complete confluence, they were established as ALI cultures by removing the culture medium from the apical side of the cultures and providing DMEM: BEGM (1:1) medium containing 50 nM retinoic acid (Sigma-Aldrich, Missouri, U.S.A.) as the ALI-growth medium from the basolateral side. The medium was changed every day, and the apical surface mucus was removed by gently washing the cultures with PBS.

Epithelial cells in culture were treated with four different conditions: (a) EETs (0.5 μg/ml), (b) EETs (0.5 μg/ml) + DNase I (3 mg/ml) (Solarbio, Beijing, China), (c) DNase I (3 mg/ml), and (d) unstimulated eosinophils (10 μl). These treatments were carried out for a duration of 24 h. After treatment, both the cultured cells and supernatant were collected and analyzed using real-time PCR and ELISA to evaluate the expression levels of eotaxin-3.

### Cytokine measurements

IL-4, IL-5, and IL-13 protein levels in frozen tissue were measured using Meso QuickPlex SQ 120 (Meso Scale Discovery, Maryland, U.S.A.) following the manufacturer’s protocol. Briefly, frozen tissues were dissociated and sliced on ice. Each 100 mg of tissue was homogenized in 1 ml of RIPA containing 10 μl/ml of PMSF. The suspension was then centrifuged for 5 min at 12,000 ***g*** and 4°C. To determine the total protein concentration in the supernatant, the BCA assay was employed. Initially, the U-PLEX plate was washed with a washing buffer containing PBS and 0.05% Tween 20. Then, 50 μl of homogenate from each sample was added to the respective wells of the U-PLEX plate. The plate was sealed and incubated with shaking for 2 h. Subsequently, the plate was washed three times with the washing buffer. Next, 25 μl of the corresponding detection antibodies were added to each well, and the plate was incubated with shaking for an additional 2 h. Finally, the plate was washed again, and 150 μl of 2× Read buffer was added per well. The MESO QuickPlex SQ 120 instrument (Meso Scale Discovery, Maryland, U.S.A.) was used to measure the protein concentration immediately.

### ELISA

Cell supernatants were assayed for CLCs (Mybiosource, San Diego, U.S.A.) and eotaxin-3 (MULTISCIENCES, Hangzhou, China) using ELISA kits following the manufacturers’ instructions. The CLCs protein detection range was 0.3–20 ng/ml, and the eotaxin-3 protein detection range was 15.63–1000 pg/ml. Samples were appropriately diluted, and 100 μl of standard or diluted sample was added to the ELISA plate. Detection agent A and detection agent B were sequentially added to each well and incubated at 37°C for 1 h. The plate was then incubated with TMB substrate at 37°C in the dark for 15 min and the reaction stopped with stop buffer. The optical density (OD) values of each well were measured using a Rayto RT-6100 (Rayto, Shenzhen, China). The corresponding concentrations were obtained from the standard curve based on the OD values, and the final OD values of the samples were calculated by multiplying the concentrations by the dilution factor.

### Statistical analysis

Statistical analysis was conducted using GraphPad Prism 9.4 software. The differences between groups were assessed using a two-tailed Student’s *t*-test, and the data were presented as mean ± SEM. Demographic factors(sex), nasal polyp status, asthma were analyzed by chi-square test. The Pearson and Spearman correlation tests were used to perform linear correlation analysis. For normally distributed data, ANOVA was applied for multiple comparisons. *P*-value of ≤ 0.05 was considered statistically significant.

## Results

### Demographic data from study subjects

Table 1 presents the clinical characteristics of the study participants. There were no significant differences in age and gender distribution between the two groups (*P*>0.05). However, patients in the eCRS group had a significantly higher prevalence of comorbidities such as polyps (100% vs. 70.6%, *P*<0.01) and asthma (30.4% vs. 8.8%, *P*=0.03). The clinical assessments also showed that patients in the eCRS group had significantly higher scores for peripheral blood eosinophils (0.33 ± 0.18 vs. 0.16 ± 0.06, *P*<0.0001), VAS score (6 ± 1.1 vs. 4.2 ± 1.1, *P*<0.01), Lund–Mackay endoscopic score (14.7 ± 3.5 vs. 10.7 ± 3.5, *P*<0.01), Lund–Kennedy CT score (8.58 ± 2.04 vs. 6.1 ± 2.6, *P*<0.01).

**Table 1:** Baseline characteristics of the participators

Characteristics	Non-eCRS (*n*=34)	eCRS (*n*=23)	*P-*value
Mean age, years	47 ± 13.61	46.63 ± 14.60	0.22
Male sex, *n* (%)	25(74%)	20 (87%)	0.57
Polyps, *n* (%)	24(70.6%)	23 (100%)	<0.01
Asthma, *n* (%)	3(8.8%)	7 (30.4%)	0.03
VAS	4.2 ± 1.1	6 ± 1.1	<0.01
Peripheral blood eosinophil (10^9^/L)	0.16 ± 0.06	0.33 ± 0.18	<0.001
Lund–Mackay	10.7 ± 3.5	14.7 ± 3.5	<0.01
Lund–Kennedy	6.1 ± 2.6	8.58 ± 2.04	<0.01

Abbreviations: eCRS, eosinophilic chronic rhinosinusitis; Non-eCRS, non-eosinophilic chronic rhinosinusitis; VAS, visual analog scale.

### Increased presence of EETs in eCRS

**Figure 1:**
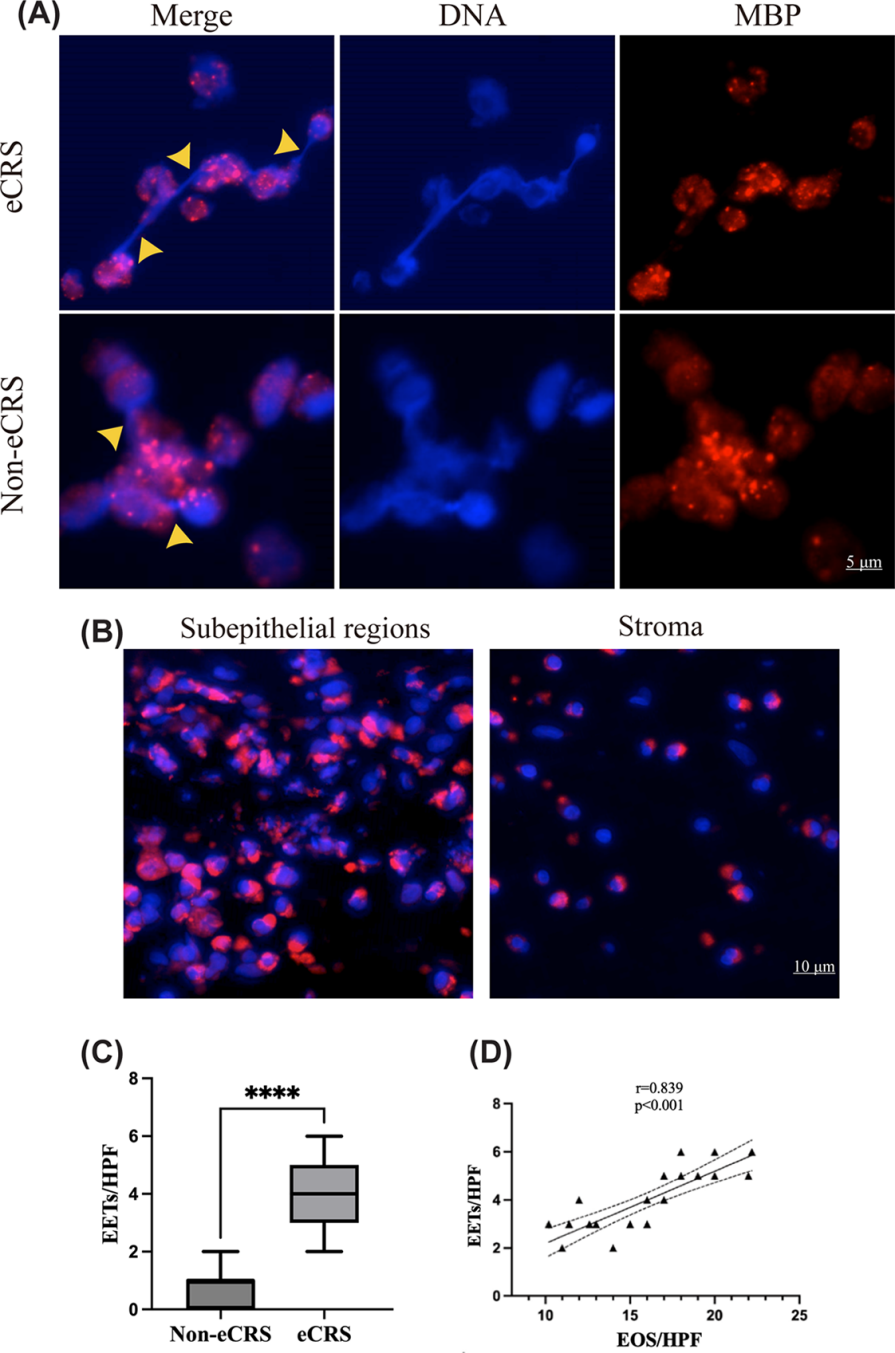
Identification of EETs in both Non-eCRS and eCRS tissues (**A**) immunofluorescent staining of MBP (red) and DNA (blue) for EETs (indicated by the yellow arrow) in the subepithelial region. The quantification of EETs in the subepithelial region of Non-eCRS and ECRS tissues is shown. Scale bar = 5 μm. (**B,C**) EETs were found mainly in the subepithelial regions, whereas intact eosinophils were observed in the stroma. Scale bar = 10 μm. Figure 1C shows that EETs formation was significantly higher in ECRS tissue than in Non-eCRS tissue (****P<0.0001). Figure 1D shows that EETs formation was significantly correlated with tissue eosinophils (*P*<0.001). The lines indicate the mean with 95% confidence interval.

Both the eCRS and Non-eCRS groups exhibited the presence of EETs. In the Non-eCRS group, extracellular DNA nets were observed in association with the granular protein MBP (Figure 1A). Eosinophils were mostly intact in the basal parts of the epithelium or stroma, and EETs formation was rare. However, in subepithelial regions with missing epithelial cells, eosinophils were frequently observed in clusters, and massive and clustered EETs were often released (Figure 1B). The eCRS group had a significantly higher number of EETs than the Non-eCRS group (3.96 ± 1.26 vs. 0.61 ± 0.55, *P*<0.0001, Figure 1C). Moreover, the formation of EETs was positively correlated with the number of tissue eosinophils (*r* = 0.8395, *P*<0.001, Figure 1D).

### EETs correlates with the disease severity

**Figure 2:**
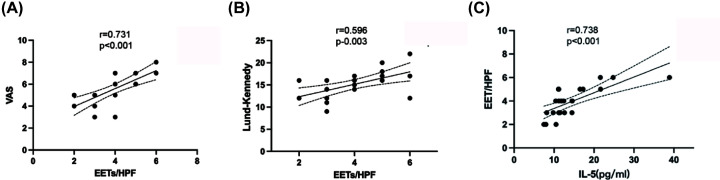
Disease severity in correlation with the extent of EETs formations in eCRS (**A**) A Pearson test was used to analyze correlations between VAS score and EETs (**B**) A Pearson test was used to analyze correlations between preoperative CT score and EETs (**C**) A Pearson test was used to analyze correlations between preoperative IL-5 protein level and EETs. The lines represent the mean with a 95% confidence interval.

EETs showed correlations with some clinical features in patients with eCRS. Our findings demonstrated a positive correlation between the number of infiltrating EETs-releasing eosinophils and the VAS score and preoperative total Lund–Mackay CT score in CRS patients (*r* = 0.731, *P*<0.001 and *r* = 0.596, *P*<0.003; Figure 2A,B, respectively). We found no significant correlation between the level of Lund–Kennedy endoscopic score and the number of EETs (*r* = 0.131, *P*=0.55). Nonetheless, we also observed no significant association between the peripheral blood eosinophils and the quantity of EETs (*r* = 0.256, *P*=0.24).

IL-5 is a critical pro-inflammatory cytokine that regulates the homeostasis and proliferation of eosinophils. The combination of IL-5 and LPS is known to induce EETs. We found that eCRS patients with higher numbers of EETs had significantly higher tissue protein levels of IL-5, and there was a positive correlation between EETs formation and IL-5 levels (*r* = 0.738, *P*<0.001, Figure 2C). Interestingly, we did not observe any correlation between EETs formation and IL-4 or IL-13 protein concentrations in the tissue (*r* = 0.2367, *r* = 0.226, *P*>0.05).

### Association between EETs formation and CLCs production

**Figure 3:**
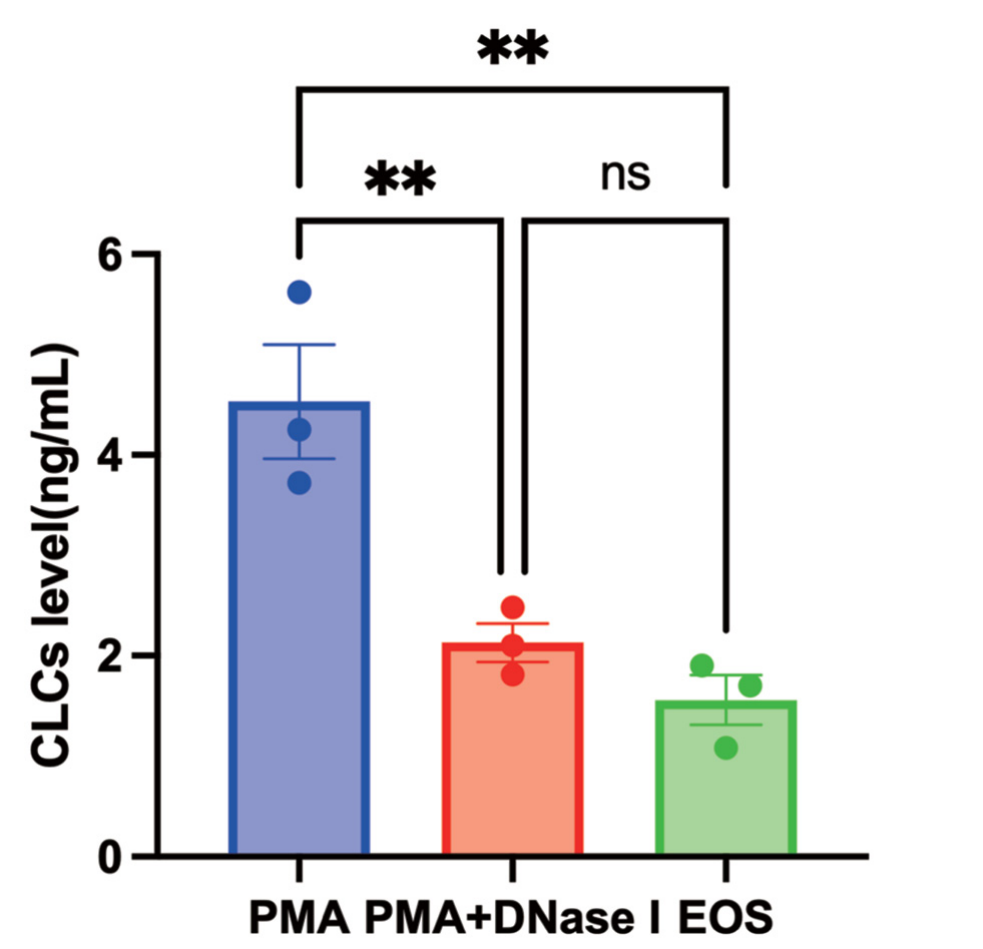
The release of EETs can increase the expression of CLCs protein Protein levels of CLCs, *n*=3; **P<0.01, ns: *P*≥0.05.

CLCs are one of the important biomarkers of eosinophilic inflammatory diseases. To further investigate the relationship between the release of EETs and the formation of CLCs, the eosinophil suspension extracted from peripheral blood is divided into three groups: the positive control group is treated with 10 ng/ml PMA, the negative control group with 10 ng/ml PMA+10U/ml DNase I, and the blank control group with no additional components. The expression levels of CLCs in the cell supernatants are detected using ELISA. The experimental results show (Figure 3) that after the addition of PMA to induce the release of EETs from eosinophils, the formation of CLCs increases (4.53 ± 0.98 ng/ml). In eosinophil cells treated with PMA+Dnase I and blank control, the CLC levels are 2.13 ± 0.34 ng/ml and 1.56 ± 0.43 pg/ml, respectively.

### EETs induce eotaxin-3 secretion by HNECs

**Figure 4:**
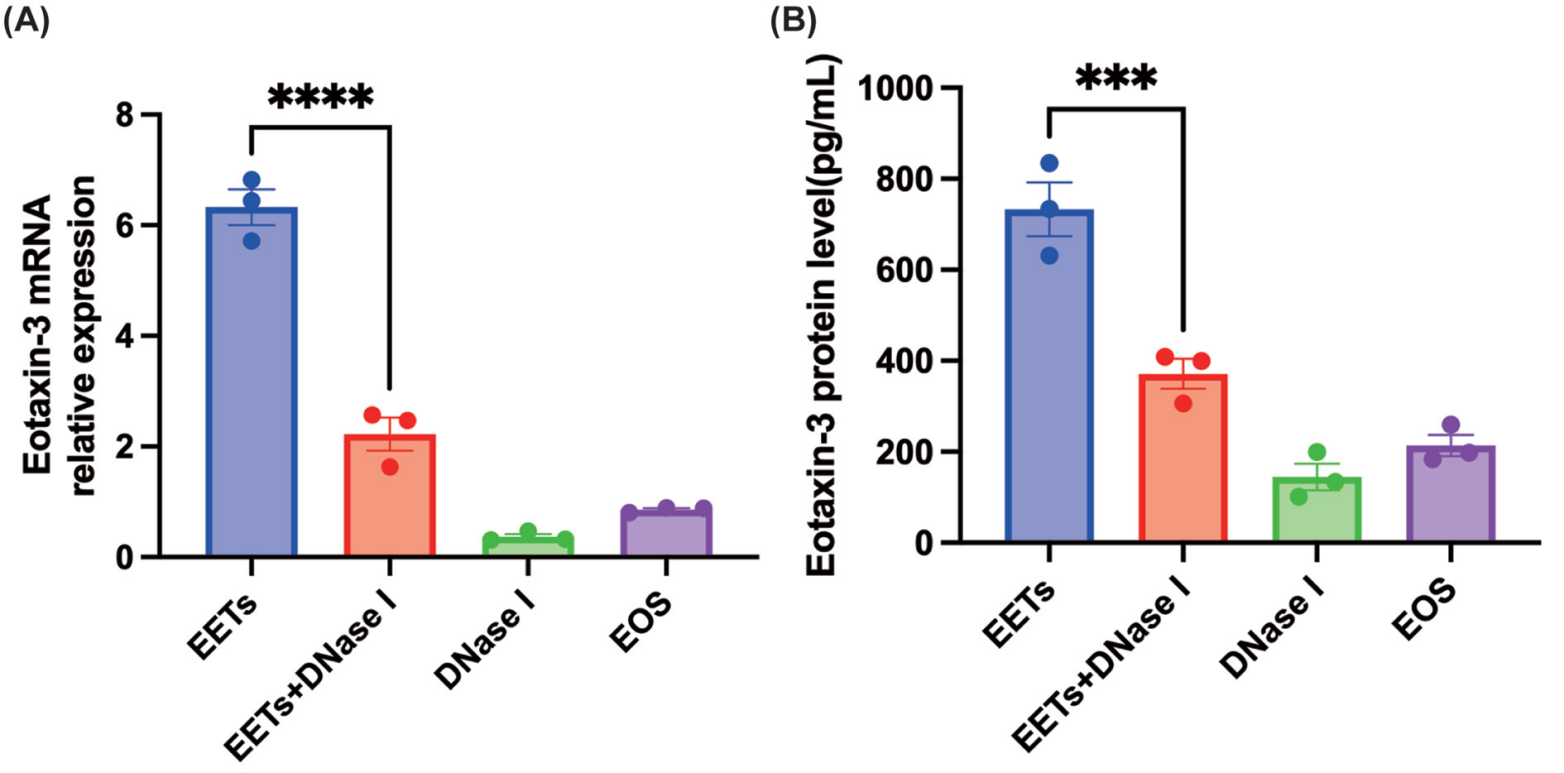
The expression levels of eotaxin-3 secreted from cultured epithelial cells stimulated with the supernatants of EETs, EETs+DNase, DNase, and unstimulated eosinophils were evaluated by real-time PCR and ELISA (**A**) Relative expression of eotaxin-3 mRNA. (**B**) Protein levels of eotaxin-3; *n*=3; ***P<0.001, ****P<0.0001.

Since the sinonasal epithelium is a major source of chemokine secretion in CRS, we investigated the functional significance of EETs by analyzing the chemokines secreted from epithelial cells. After treating cultured epithelial cells with EETs, EETs+DNase, DNase, or unstimulated eosinophils, we analyzed the expression levels of eotaxin-3 in the cell supernatants using real-time PCR and ELISA. Our results showed that exposure to EETs in air liquid culture significantly increased mRNA expression of eotaxin-3 compared with the other treatment groups (Figure 4A). Similarly, cells stimulated with EETs exhibited increased levels of eotaxin-3 protein (733.03 ± 101.98 pg/ml) in the supernatants, whereas their levels were not increased in cells treated with EETs+DNase I, DNase I, or unstimulated eosinophils (371.51 ± 56.74 pg/ml, 144.6 ± 50.10 pg/ml, and 213.8 ± 40.52 pg/ml) (Figure 4B).

## Discussion

Eosinophils, a minor subset of leukocytes, can contribute to eosinophilic inflammation by actively inducing cytolytic cell death that results in the release of EETs and other cellular contents. Our study identified that EETs are predominantly present in subepithelial regions and largely depend on the presence of tissue eosinophils. Additionally, we found a significant increase in the number of EETs in patients with severe CRS, which strongly correlates with IL-5 levels. Our findings suggest that the EETs generated CLCs can contribute to eosinophilic inflammation in HNECs. Eotaxin-3, an epithelium-derived cytokine, plays a vital role in eosinophil chemotaxis. However, it remains unclear whether EETs stimulate the release of eotaxin-3 from epithelial cells. Our study is the first to demonstrate that EETs induce the secretion of eotaxin-3 by epithelial cells, which may further recruit eosinophils and contribute to the development of eosinophilic inflammation.

Several earlier research studies have shown the existence of EETs in a variety of eosinophilic illnesses [5]. EETs are developed in nasal polyp tissue, primarily at subepithelial locations with epithelial barrier abnormalities [7]. Our study further indicates that some eosinophils in the inflamed nasal mucosa of both eCRS and non-eCRS patients generate EETs, which are composed of released filamentous chromatin structures in association with eosinophil granule proteins in the extracellular matrix. In the group analysis, the eCRS group had a considerably higher number of EETs than the non-eCRS group, and the development of EETs was detected more frequently in the presence of numerous tissue eosinophils. Although EETs formation is associated with the number of eosinophils in the tissue, no correlation exists with the number of eosinophils in the blood. This discrepancy may arise from the fact that local eosinophil activities at the tissue level, such as activation and migration, frequently transpire without a concomitant increase in blood eosinophils. EETs were primarily found in subepithelial regions and were related with disturbance of the mucosal barrier. Many eosinophils deposited in the basal portions of the epithelium or deep in the stroma were discovered to have granules that were largely intact. EETs were reported as being capable of entrapping and killing *S. aureus*, which may explain why they were predominantly found in epithelium defects in direct contact with *S. aureus* [14]. Our results suggest that EETs, regardless of phenotype, are produced by tissue eosinophils and may contribute to the barrier function of the nasal mucosa.

ECRS is often associated with more severe symptoms and treatment-resistant cases compared with non-eCRS. So far, there is still no consensus on how to characterize tissue eosinophilia, mostly due to the uneven distribution of eosinophils within the tissue. To establish a biomarker for endotyping, it is crucial to determine if there is a difference in clinical outcomes between endotypes. In this study, we employed a series of clinically relevant tests to assess the correlation between EETs and disease severity. Our findings suggest that an increased number of EETs correlates with a greater disease burden, as indicated by higher VAS and CT severity scores. This indicates that EETs may be useful in evaluating disease severity and even predicting the rate of relapse in eCRS patients.

The association between elevated IL-5, eosinophils, and CRS has been well-established for many years [15]. IL-5 is a critical factor for the differentiation, migration, activation, and survival of eosinophils, and its receptor expression in eosinophils has given it a target for CRS therapy [16]. In addition, IL-5 and numerous other mediators stimulate eosinophils to produce toxic granule proteins via secretion mechanisms and cytolytic EETosis. Our studies have established a correlation between the formation of EETs and elevated levels of the cytokine IL-5, showing the significance of IL-5 expression in the generation of EETs. Currently, no studies have found that IL-4 and IL-13 are involved in the activation of EETs. Our research results also found no clear correlation between EETs and the expression levels of these two cytokines. Therefore, IL-4 and IL-13 may not be involved in the release process of EETs.

CLCs, a hallmark of eosinophilic inflammation, have been reported in several Th2 inflammatory disorders, including asthma, CRS, and other eosinophil-associated diseases [17] CLCs have been shown to induce the release of the proinflammatory cytokine IL-1β, which plays a role in eosinophilic immune functions [18]. Several studies have shown that CLCs expression is upregulated in eosinophilic CRSwNP, and it can serve as a potential marker for identifying patients who may respond to glucocorticoid treatment and have a higher risk of recurrence [19,20]. Another study showed that EETosis in CRSwNP tissue is associated with CLCs deposition [4]. Bachert et al recently discovered that CLCs can induce the recruitment of neutrophils and promote the release of cytokines like GM-CSF, TNF-α, and IL-1, which could potentially enhance neutrophil function [8]. In the present study, we found that increased CLCs formation was present after stimulation of PMA in eosinophil. Additionally, we found that inhibition of EETs by DNase I can reduce the production of CLCs. These findings indicate that reducing EETs formation could potentially be a new therapeutic approach for treating CRS by reducing CLCs formation.

The sinonasal epithelium responds to mucosal pathogens by producing chemokines, which recruit immune cells and initiate mucosal inflammation. The CXC chemokine family is known to be chemotactic for neutrophils, while the CC chemokine family is chemotactic for eosinophils. A recent study found that epithelial cells stimulated with NETs produced increased levels of various chemokines, including eotaxin-3, a cytokine that induces chemotaxis of Th2 lymphocytes, eosinophils, and basophils [9]. Significantly elevated levels of plasma eotaxin-3 have been observed in patients with tissue eosinophils greater than 55 per HPF, which has been proposed as a potential marker for disease recurrence [21]. Our study demonstrated that EETs can also have proinflammatory effects on HNECs, contributing to further recruitment of eosinophils through eotaxin-3 secretion. Moreover, we found that inhibition of EETs by DNase treatment decreased eotaxin-3 production. Although previous studies have demonstrated that IL-13-induced 15-lipoxygenase 1 (15LO1) promotes intracellular and secreted CCL26 expression via activation of the ERK pathway, no direct evidence of the relationship between EETs and 15LO1 has been established [22]. In addition, Choi et al. discovered that mice injected with EETs had significantly elevated levels of eotaxin-2 and a higher proportion of IL-5 or IL-13 producing ILC2s in the lungs [13]. This suggests that EETs have the ability to induce ILC2s to produce IL-13, which could subsequently lead to eotaxin-3 secretion by HNECs.

The current study has several limitations that require further investigation. Firstly, the *in vivo* pathophysiological role of EETs was not verified. Although a previous study indicated elevated EET formation in ovalbumin-challenged mice, it remains unknown whether EETs have an impact on CRS mice. Secondly, it is unclear whether the identified EETs are a direct result of epithelial barrier impairment or partly accountable for causing epithelial damage. Future research is necessary to determine the stimuli responsible for EETs formation and to elucidate the underlying molecular mechanisms in CRS.

Although EETs were initially discovered as an alternative mechanism for eosinophils to combat pathogens, their prolonged accumulation can lead to host tissue damage. Our study revealed a higher number of EETs in the nasal tissue of patients with eCRS compared to those without eCRS. Additionally, we found that EETs can prompt the development of CLCs and trigger the secretion of chemokines that further facilitate eosinophil infiltration into the nasal mucosa, causing sustained inflammation. Our findings suggest that EETs hold potential as a biomarker for endotyping and as a therapeutic target for eCRS. However, further research is required to comprehensively understand the molecular mechanisms behind EETs induction and their contribution to the pathogenesis of eCRS.

## Data Availability

The datasets that were used and/or analyzed in the current study are available upon reasonable request from the corresponding author.

## Abbreviations

15LO1: 15-Lipoxygenase 1
CLC: Charcot–Leyden crystal
CRS: chronic rhinosinusitis
CRSsNP: chronic rhinosinusitis without nasal polyps
CRSwNP: chronic rhinosinusitis with nasal polyps
ECRS: eosinophilic chronic rhinosinusitis
EETs: eosinophil extracellular traps
EOS: eosinophils
HNEC: human nasal epithelial cell
NET: neutrophil extracellular trap
Non-eCRS: non-eosinophilic chronic rhinosinusitis
NP: nasal polyp
